# Seek the Spindle Tumor in Submandibular Space

**Published:** 2019-05

**Authors:** Deviprasad Dosemane, Urmila Khadilkar, Meera Khadilkar, Fayis Anwar

**Affiliations:** 1 *Department of Otorhinolaryngology, Kasturba Medical College, Mangalore, Manipal Academy of Higher Education. India.*; 2 *Department of Pathology, Kasturba Medical College, Mangalore, Manipal Academy of Higher Education. India.*

**Keywords:** Immunohistochemistry, Inflammatory myofibroblastic tumor, Spindle neoplasm, Submandibular

## Abstract

**Introduction:**

Submandibular region is surgically significant in the head and neck field and a mass in this region may have multiple differential diagnoses.

**Case Report:**

An elderly man came with a single 4×2.5 cm swelling in the neck on the right side, just below the lower jaw, since one month. Ultrasound showed an irregular heterogeneous hypoechoic lesion in the right submandibular space measuring 37×23 mm with mild internal vascularity. The submandibular gland appeared separate but compressed. Fine needle aspiration was suggestive of spindle cell neoplasm. The swelling was excised under general anesthesia. By histopathological examination, the lesion was diagnosed as anaplastic lymphoma kinase-negative inflammatory myofibroblastic tumor (IMT) based on focal immunoreactivity with cyclin D1. The patient then received radiotherapy 60 Gray divided into 30 fractions over 6 weeks. The case had no evidence of recurrence or residual disease six months post-surgery.

**Conclusion:**

Tumefactive spindle-cell lesion in the head and neck can comprise inflammatory conditions, benign and malignant neoplasms or borderline neoplasms, such as nodular fasciitis and IMT. The definitive histologic diagnosis of IMT helps in tailoring the treatment modality based on its locally aggressive biologic potential.

## Introduction

The submandibular region is surgically significant in the head and neck field; with the presentation of isolated submandibular mass. Differentials for the same are the pathologies of the salivary gland, lymph node, vascular, soft tissue, and neuronal lesions. Submandibular gland lesions include sialolithiasis, sialolithiasis, as well as benign and malignant tumors (1,2).

## Case Report

A 74-year old man, farmer, came to Ear Nose Throat Outpatient Department with a swelling in the neck on the right side, just below the lower jaw, since one month. It was insidious in onset and gradually progressive. There was no fever, pain over the swelling or change in the size of the swelling during the meals. The patient was a known case of coronary artery disease on a pacemaker. 

**Fig1:**
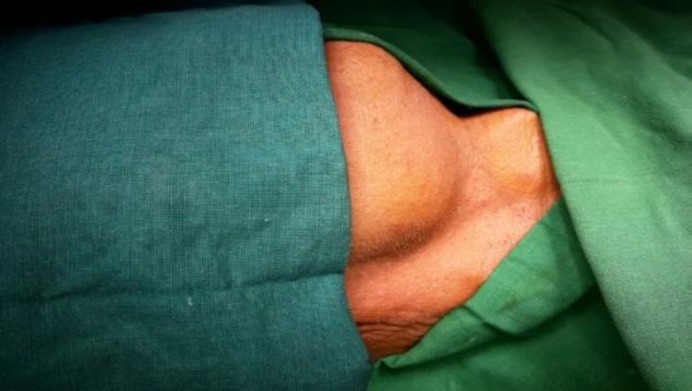
Preoperative photograph demonstrating right submandibular swelling

The examination revealed a single 4×2.5 cm swelling in the neck below the right lower margin of the mandible extending anteriorly 3 cm from the midline to the right, posteriorly 3cm from the mastoid tip, superiorly till the lower margin of ramus of the mandible, and inferiorly 2 cm below the lower margin of the ramus of the mandible (Fig.1). The palpation of the swelling revealed a nontender and firm to hard mobile mass with no local rise in the temperature. The surface was smooth and the skin over the swelling was pinchable. The swelling was neither bimanually palpable nor ballotable. 

**Fig 2:**
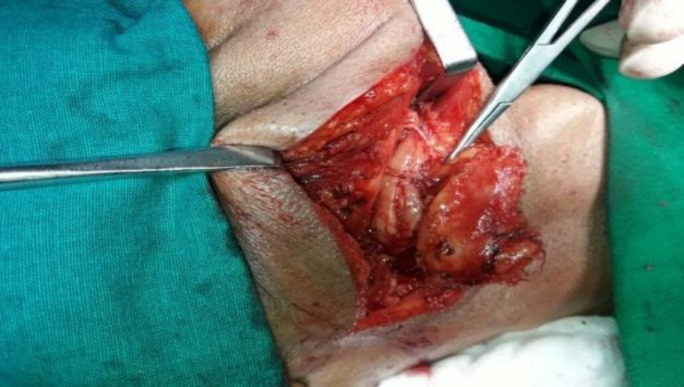
Intraoperative photograph of excision of swelling

The ultrasound showed an irregular heterogeneous hypoechoic lesion in the right submandibular space measuring 37×23 mm with mild internal vascularity. The submandibular gland appeared separate but compressed. Few small subcentimeter-sized right level II, level III, level V, left level II nodes, and likely reactive nodes were also noted. Fine needle aspiration was suggestive of spindle cell neoplasm. Lab parameters were within normal limits. The swelling was excised under general anesthesia. Intraoperatively, a 3.5×2.5 cm mobile swelling was identified in the right submandibular space, separate from the submandibular gland, superior and lateral to it, and suspected to be arising from a thin nerve lateral to mylohyoid (Fig. 2). 

**Fig 3:**
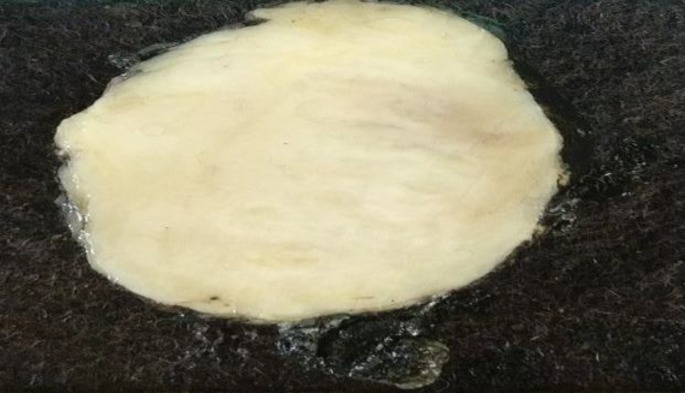
Well-circumscribed tumour with grey-white cut surface

No lymph nodes were identified. The specimen was removed in toto and sent for histopathological examination.Grossly, it was an unencapsulated lesion covered by adipose tissue (Fig.3). 

 Microscopy showed fascicles of spindle cells, scattered myofibroblastic cells with hyperchromatic nuclei and nucleoli against a background of lymphocytes, plasma cells, and scattered lymphoid follicles. In addition, brisk mitoses were observed in the spindle cells. Lack of zonation ruled out the diagnosis of nodular fasciitis. A differential diagnosis of spindle cell carcinoma was ruled out with immunohistochemistry (IHC). 

**Fig 4:**
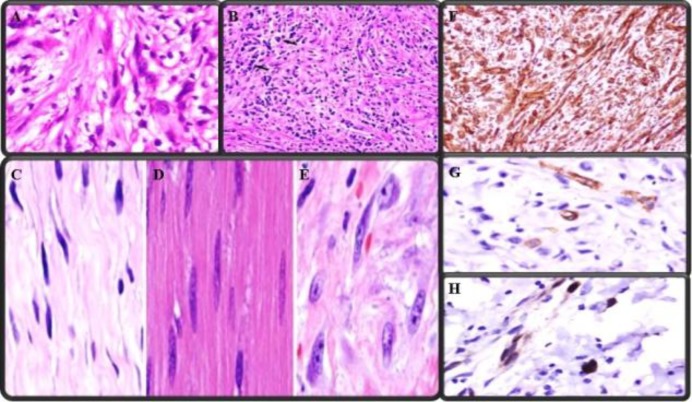
Microscopic photographs showing A.myofibroblasts, B.inflammatory cells, in the background of C.spindle cell, D.fibroblasts E.myofibroblasts, F.diffuse vimentin positivity, G. focal SMA positivity, H. focal cyclin-D1 positivity

A panel of IHC showed diffuse and strong immunoreactivity for vimentin in spindle cells and focal positivity for smooth muscle actin. The spindle cells were negative with anaplastic lymphoma kinase (ALK), cytokeratin, desmin, S100, and CD117. Focal reactivity with cyclin D1 favored a diagnosis of inflammatory myofibroblastic tumor (IMT) (Fig 4 A-H). The case then received radiotherapy 60 Gray divided into 30 fractions over 6 weeks. The patient had no evidence of recurrence or residual disease six months post-surgery.

## Discussion

Among the soft tissue tumors, IMT is a rare entity, which was first described in 1937. It falls under the group of intermediate borderline malignant tumors, which often recur and rarely metastasise. It commonly affects the abdomen, lungs, soft tissue, skin, genital system, and mediastinum. Manifestation in the head and neck is rare, such as the submandibular space that is reported in the present study (3). Chen et al. reported ten cases in the head and neck in a course of 9 years, which comprised four in maxillary space (40%), two in buccal space and parotid (20% each), as well as one in the neck and post aurem (10% each). Sometimes, it can synchronously present at multiple sites (4).

IMT was formerly called “pseudotumor” due to the expansive nature and its radiological similarity to malignancy. World Health Organization designated it as an intermediary soft tissue tumor, encompassing spindle cells, differentiated myofibroblasts, and abundant inflammatory cells, such as plasma cells plus or minus lymphocytes. The head and neck lesions are frequent in children and young adults with a male preponderance (5). 

IMT has been described in the maxillary sinus, parapharyngeal space, as well as epiglottis and oral cavity. The gingiva, tongue, buccal mucosa, as well as the mandible and submandibular gland, are the frequent sites of involvement in the oral cavity (6). The etiopathogenesis in spite of being uncertain has been attributed to the factors, such as ALK gene rearrangement, viruses, including Epstein-Barr virus and human herpes virus, immunoglobulin G4, trauma, chronic inflammation, and autoimmune process (8,7).

IMT is classically diagnosed by histopathological examination. Immunohistochemistry typically indicates positivity with vimentin, as well as smooth muscle actin. The salient expression of ALK on IHC is associated with the rearrangements of ALK observed in most of the IMT cases (8). Histologically, IMT consists of myofibroblastic spindle cells plus noticeable infiltration of plasma cells and lymphocytes. Three histological patterns have been proposed, including myxoid (1), vascular, inflammatory areas, such as nodular fasciitis compact spindle cells plus intermingled inflammatory cells (2), such as fibrous histiocytoma; and dense plate-like collagen, such as a scar or desmoids (3). Usually, individual tumors contain various proportions of these patterns (9). In the present study, IMT manifested in an elderly male at an uncommon site of submandibular space infiltrating the surrounding fat. 

Immunoreactivity was also atypical because it was ALK-negative. The most common differential in the head and neck, especially oral cavity and larynx, is sarcomatoid carcinoma, which was ruled out by negative immunoreactivity with cytokeratin, strong immunoreactivity with vimentin, and focal positivity with actin and cyclin-D1 that helped in the diagnosis of IMT (10,11).

The treatment of head and neck IMT depends on pathological subtype, tumor site, amount of local infiltration, and the probability of complete removal. Steroids and surgical resection are the modalities of treatment in combination with radiation. Complete surgical resection is encouraged for surgically accessible lesions (12,13). 

IMT in the oral cavity may be mixed up with other malignant tumors on clinical, radiographic, and histologic appearance. Therefore, it is necessary to distinguish them so as to offer better guidelines for management and outcome (14).

## Conclusions

Tumefactive spindle-cell lesions in the head and neck can comprise inflammatory conditions, such as Immunoglobulin G4 disease, benign neoplasms, such as fibromas, malignant neoplasms, such as sarcomatoid carcinomas or borderline neoplasms, such as nodular fasciitis and IMT. The definitive histologic diagnosis of IMT helps in tailoring the treatment modality based on its locally aggressive biologic potential.
